# Association between auditory pathway efferent functions and genotoxicity in young adults

**DOI:** 10.1590/S1808-86942011000100018

**Published:** 2015-10-19

**Authors:** Andressa Boer Fronza, Daniele Coronel Menna Barreto, Tânia Maria Tochetto, Ivana Batrice Mânica da Cruz, Aron Ferreira da Silveira

**Affiliations:** 1 Santa Maria Federal University. Speech therapist; 2 Santa Maria Federal University. Speech therapist; 3 São Paulo Federal University. Associate professor of the speech therapy course, Santa Maria Federal University; 4 University of California, US. Adjunct professor, Santa Maria Federal University; 5 Santa Maria Federal University. Full professor in the Morphology Department, Santa Maria Federal University

**Keywords:** hearing, genotoxicity, smoking, efferent pathways

## Abstract

Efferent auditory pathways modulate outer hair cells of the cochlea, protect against noise, and improve the detection of sound sources in noisy environments. Genotoxicity is DNA damage.

**Aim:** To study the association between auditory pathway efferent functions with genotoxic markers. The study also considered smoking and gender as two main variables.

**Methods:** A prospective-clinical, quantitative, cross-sectional, contemporary study. The function of efferent auditory pathways and genotoxicity tests in 60 healthy young subjects were assessed.

**Results:** The mean age of subjects was 24.86 years +/- 3.68 years; there were 30 males and 30 females, 15 of each gender smokers and 15 non-smokers. Male smokers had a higher incidence of DPOEA suppression effect at 2000 and 6000 Hz in the left ear; female smokers had a higher prevalence of complaints of difficulty to hear in noisy environments; smokers and women had a higher mean DNA damage; subjects with complaints of hearing loss and tinnitus had higher genotoxicity.

**Conclusions:** In young normal-hearing adults that complain about efferent auditory pathways functions, such as tinnitus and hearing impairment, there are possible associations with genotoxicity; interactions between gender and smoking are considered.

## INTRODUCTION

The auditory system consists of afferent and efferent auditory pathways that operate in an integrated manner. Efferent pathways consist of the medial and lateral olivocochlear bundles, which differ anatomically and physiologically to coordinate the independent function of both ears.^1^

Functions of the efferent auditory pathways include modulating the outer hair cells of the cochlea, decreasing the action potential of the cochlear nerve for protection against noise, locating sources of sound, and improving sound detection in noisy contexts.^1^^-^^2^ A study^3^ reported that efferent pathways are less efficient in patients with tinnitus.

The medial olivocochlear tract of the efferent system modulates the movements of the outer hair cells by releasing acetylcholine into the synaptic cleft.^4^

Efferent auditory pathways can be evaluated by applying a contralateral acoustic stimulus and simultaneously measuring otoacoustic emissions (OAEs). The OAE suppression effect by contralateral noise is used frequently in clinical and research settings because it assesses efferent pathways quickly and non-invasively.

Living cells in the human body require an adequate supply of oxygen and nutrients to function properly, which depend on structurally and functionally intact heart and blood vessels.

Adequate supply of nutrients and oxygen result in the production of energy for organisms to carry out their metabolic activities. Energy is produced in mitochondrial respiratory processes. About 5% of the oxygen that is breathed in generates active oxygen species (free radicals) rather than producing energy (adenosine triphosphate or ATP). Free radicals react strongly with other cell molecules, and if left uncontrolled, may affect physiological processes and cause disease.^5^

Organisms control free radicals with an enzymatic antioxidant system, which transforms free radicals (superoxide and hydrogen peroxide or oxygenated water) into water molecules. Aside from this endogenous system, antioxidant molecules from fruit and vegetables help defend our organism against free radicals. When free radicals are produced in more quantity than exogenous and endogenous antioxidant control mechanisms can control, oxidative stress develops. Oxidative stress damages important molecules in the body, such as cell membranes (by lipid peroxidation) and mutations in deoxyribonucleic acid (DNA). These changes are described as genotoxicity. Thus, increased genotoxicity indicates that there is more oxidative stress in the body.

Research has suggested that oxidative stress, which is caused by several risk factors including smoking, may affect hearing.^6^, ^7^, ^8^ Smoking increases carbon dioxide and nicotine levels, which in turn increase the amount of free radicals. Evidence suggests that uncontrolled production of these molecules causes endothelial dysfunction, which may result in vasoconstriction of blood vessels, and increase the incidence of thrombotic occlusion of vessels that irrigated auditory organs.^9^^-^^10^

It is therefore relevant to study a possible association between auditory efferent function and genotoxicity (a marker of oxidative stress) in normal-hearing subjects. Intervening factors, such as smoking or gender, may be relevant for the abovementioned association; smoking tends to increase oxidative stress and gender may be related with metabolic (hormonal) and behavioral differences among individuals.

Therefore, the purpose of this study was to study associations among auditory efferent pathway function as assessed by suppression of distortion product evoked OAEs (DPOAEs), self-reported hearing complaints, and genotoxicity markers of oxidative stress. This study also used smoking and gender as the main intervening variables.

## MATERIAL AND METHOD

A quantitative cross-sectional contemporary prospective clinical study was carried out to analyze auditory efferent pathway function based on the suppression effect of DPOAEs and self-reported hearing difficulties, tinnitus, hearing difficulty in noisy environments, difficulty in understanding speech in groups, and discomfort with loud sounds (complaints related with auditory efferent functions) in 60 young adult volunteers aged from 18 to 32 years. These volunteers were selected randomly from a sample of 1,024 subjects that had been enrolled in a research project: “Nutrigenetics and Smoking: In Vivo and Ex Vivo Studies of Diet and Physical Activity on the Modulation of Pro-Oxidative Effects of Smoking.” The institutional review board approved the study project (number 0146.0.243.000-07). Genotoxicity markers were also investigated in the study sample.

Inclusion criteria were having normal auditory thresholds,^11^ the presence of DPOAEs,^12^ and for smokers, having smoked more than 100 cigarettes within the last 90 days.^12^

All participants signed a free informed consent form. The study procedures consisted of:

a) *A clinical history*: comprising questions for identification, personal and family medical history, smoking habit, number of cigarettes smoked daily, cigarette brand, duration of exposure to tobacco, complaints of hearing difficulties, tinnitus, hearing difficulty in noisy environments, difficulty to understand speech in groups, and discomfort with loud sounds.
b) *Basic Evaluation of Hearing*:
  - Visual inspection of the external acoustic canal;
  - Pure tone audiometry - air conduction at 250, 500, 1000, 2000, 3000, 4000, 6000 and 8000 Hz, and bone conduction at 500, 1000, 2000, 3000 and 4000 Hz, if necessary, in a descending run^13^. This test was done with a two-channel type I Fonix model FA-12 audiometer with Telephonics TDH-39P in-ear phones, ISO 389-1991 calibrated. Subjects were considered normal-hearing when the mean airway threshold pure tones at 500, 1000 and 2000 Hz ranged from 0 to 25 dB.^11^
  - Speech Recognition Threshold study (SRT) with disyllables;
  - Speech Recognition Index with monosyllables. Results were normal if within the 92 to 100% correct answer rate, where subjects have no difficulties understanding speech.^14^
  - Acoustic Immittance Measures: tympanometry and acoustic reflexes (contralateral and ipsilateral). An Interacoustics AZ7 middle ear analyzer with TDH-39 phones and an MX-41 pad, and a 220Hz tone probe at 70 dBHL, ISO 389-1991 calibrated, was used for acoustic immittance testing. Acoustic reflexes were studied at 500, 1000, 2000 and 4000 Hz. Subjects were required to have a type A tympanometric curve, indicating normal mobility of the tympanic-ossicular system,^14^ and stapedial reflexes.^15,^ ^16^
    Results were annotated in a standard form.
c) *Measurement of DPOAEs and verification of the DPOAE suppression effect*: recording of DPOAEs was done in an acoustic booth, using an Interacoustics/Audiotest Clinical Otoread device.
  Two pure tones (F2/F1=1,22 ratio) were used for measuring the DPOAEs (2F1-F2); F1 was presented at 65 dBSPL and F2 was presented at 55 dBSPL. DPOAEs were measured at 1500, 2000, 3000, 4000, 5000 and 6000 Hz. DPOAEs were considered as present when the signal-tonoise ratio was ≥ 6 dB.
  DPOAEs were measured first in the absence and then in the presence of noise in the contralateral ear.
  Contralateral white noise generated by a digital two-channel audiometer (Fonix, model FA-12 type I and Telephonics TDH-39P in-ear phones at 60 dBHL) was used as a suppressive acoustic stimulus.
  Earphones were placed over the contralateral ear before recording DPOAEs to avoid handling of the DPOAE probes.
  Contralateral suppression of DPOAEs was calculated by subtracting the DPOAE response level with contralateral acoustic stimulation from DPOAEs responses without contralateral acoustic stimulation.^17^ Negative values indicated DPOAE suppression, and zero or positive values indicated absence of suppression; larger negative numbers indicate more activity of the medial olivocochlear system.
  The frequencies 2000, 3000, 4000, 5000 and 6000 Hz were selected for an analysis of the suppression effect. An analysis for each ear was also done; in this case, suppression was considered as present when it manifested in at least three of five tested frequencies.
d) *Genotoxicity testing*: the comet assay genotoxicity test and micronuclei analysis are internationally accepted for evaluating DNA damage.^18^^-^^19^ Biological samples (peripheral blood and mouth swabs) were collected for these tests by a trained pharmacist. Both genotoxicity tests were run.

### Genotoxicity testing - comet assay

**Figure 1:**
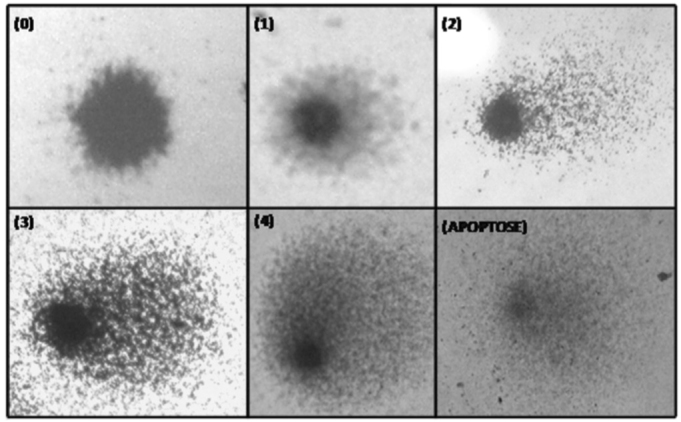
Classification of DNA damage in study subjects. Higher numbers indicate more damage. Class 0 are undamaged cells and class 4 are the most damaged cells. - (the image has no key)

The comet assay was carried out according to the method proposed by Singh et al (1988)^20^ and modified by Collins et al (1995).^21^ Two slides were prepared for each subjects. Slides were studied using an Olympus® model CX40 binocular optic microscope at 400x magnification. One hundred cells were counted for each sample (50 per slide). The five classes for classifying the comet are those proposed by García (2004);^22^ they are shown on Figure 1.

A DNA damage index was defined based on the results; it indicates genotoxicity by dividing the total number of damaged nuclei by the total number of undamaged nuclei. Each subject had two slides: 50 nuclei were counted per slide, and two independent observers analyzed both slides. The damage index was the mean number obtained from both observers for 100 nuclei per subjects. Results were recorded on a standard form.

### Genotoxicity test - micronuclei analysis

The presence of micronuclei was studied in oral mucosa cells. Epithelial cells were collected by asking subject to chew softly on their cheeks to release cells, and then to take samples from the cheek surface with a wood spatula. Subjects then placed the spatula in a conical test tube containing 3 to 5 ml of 0.9% NaCl saline solution at 6°C.

Test tubes were then centrifuged at 1,000 rpm during 10 minutes. The saline solution was changed to wash the cells, taking care to avoid removing the cells from the lower part of the tube. The solution with cells was homogenized with a Pasteur pipette, and again centrifuged. The cell washing/centrifuging cycle was done twice. Next, 1 ml of saline solution was added, homogenized, and spread over two slides (500μL in each) to have a backup copy for each subject. Slides were dried at ambient temperature, and then stained using the Panoptic Staining Kit (Laborclin®).

**Figure 2:**
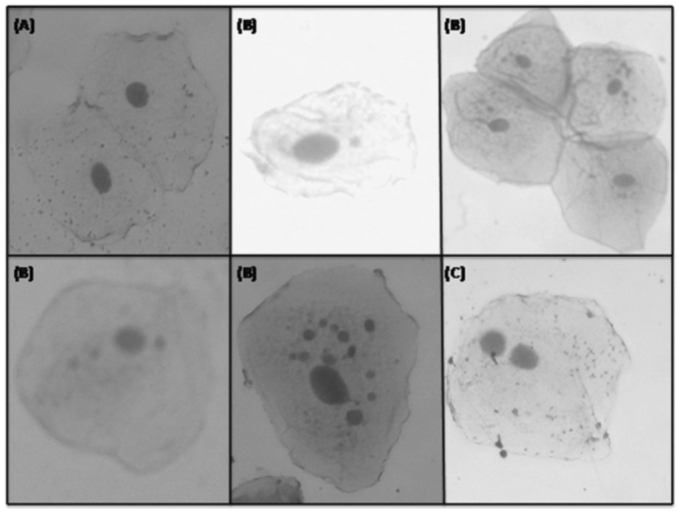
Test of micronuclei in study subjects: (A) Cell without micronuclei; (B) Cells with one or more micronuclei; (C) Binucleated cell. - (the image has no key).

After dry, the material was studied using an Olympus® model CX40 binocular optic microscope at 400x magnification for micronuclei counting and further data analysis. Figure 2 presents the micronuclei count and classification for 1,000 cells (500 per slide). Results were recorded on a standard form.

### Statistical analysis

Statistical analysis was made after data gathering. The SPSS® version 13.0 software was used for this purpose.

The tests consisted of parametric analyses (one-way analysis of variance and multivariate analysis), Bonferroni's post hoc test if there were more than three parameters in the variable, and Student's t test if there were only two parameters. The chi-square test of Fisher's exact test were applied for categorical variables. Significant associations had p ≤ 0.05.

## RESULTS

The mean age of volunteers was 24.86±3.68 years. There were 30 male and 30 female subjects (n=60). Of these, 15 of each gender were smokers and 15 were non-smokers.

### Auditory efferent functions

Table 1 presents the hearing complaints as related to efferent pathways, and table 2 presents suppression of DPOAEs in 60 subjects comprising the study sample.

**Table 1:** Hearing complaints as related to efferent pathways in young normal-hearing adults

Hearing complaints	Presence N (%)	Absence N (%)
Hearing difficulty	7 (11,7)	53 (88,3)
Tinnitus	10 (16,7)	50 (83,3)
Difficulty to hear in noisy environments	28 (46,7)	32 (53,3)
Difficulty to understand speech in groups	44 (73,3)	16 (26,6)
Discomfort with loud sounds	28 (46,7)	32 (53,3)

**Table 2:** DPOAE suppression effect in young normal-hearing adults

DPOAE suppression effect frequency/ear	Presence N (%)	Absence N (%)
2000 Hz/ear - right side	27 (45,0)	33 (55,0)
4000 Hz/ear - right side	30 (51,7)	28 (48,3)
6000 Hz/ear - right side	20 (34,5)	38 (65,5)
2000 Hz/ear - left side	26 (43,3)	34 (56,7)
4000 Hz/ear - left side	20 (33,3)	40 (66,7)
6000 Hz/ear - left side	18 (30,5)	41 (69,5)

DPOAEs - distortion product otoacoustic emissions; Hz - Hertz.

An analysis of the frequency of suppressed DPOAEs per ear (responses in at least three of five tested frequencies) showed that 19 subjects (31.7%) presented the DPOAE suppression effect in the right ear and 41 (68.3%) did not. In the left ear, 16 subjects (26.7%) presented the DPOAE suppression effect, while 44 subjects (73.3%) did not.

There was an interaction between gender and smoking in relation to certain altered functions of auditory efferent pathways.

Non-smoking males had a lower rate of DPOAE suppression at 2000 Hz in the left ear (n=05; 33.3%) compared to smokers (n=12; 80%). The difference was statistically significant (p=0.010). DPOAEs suppression occurred in 42.9% subjects (n=6) at 6000 Hz in the left ear of male smokers; it occurred only in 6.7% of non-smoking subjects (n=1) (p=0.023). There were no significant differences in the suppression effect in male smokers and non-smokers at other frequencies.

There were no statistically significant differences in hearing difficulties, tinnitus, hearing difficulties in noisy environments, difficulty to understand speech in groups, and discomfort with loud sounds among male smokers and non-smokers.

**Figure 3:**
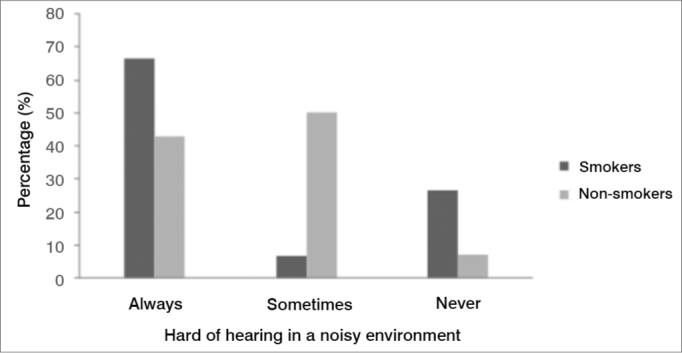
Complaints of hearing difficulty in noisy environments in normal-hearing young adult females, smokers and non-smokers (p=0.03). - (the image has no key).

Except for the complaint hearing difficulty in noisy environments, all parameters were similar among female smokers and non-smokers. Figure 3 shows that female smokers had a higher rate of hearing difficulty in noisy environments compared to female non-smokers.

### Measures of genotoxicity

The genotoxicity indicators in this study, evaluated by multivariate analysis, were compared among male and female smokers and non-smokers. Table 3 presents the genotoxicity indicator values, as follows: DNA damage rate as assessed using the comet assay, the presence of cells with one, two or three micronuclei, and the presence of cells with at least one micronucleus (sum of cells with micronuclei). Only the results of DNA damage rate (comet assay) and the sum of cells with at least one micronucleus were used for comparison purposes.

**Table 3:** Comparison of genotoxicity indicators according to gender and smoking habit

Genotoxicity	T/NT	Gender	Mean	±SD
DNA damage - comet assay	T	Male	43,90	28,25
		Female	67,80	36,47
		Total	55,85	34,28
	NT	Male	32,00	15,76
		Female	44,86	37,66
		Total	38,43	29,11
Total number of micronuclei	T	Male	47,30	32,10
		Female	56,03	33,96
		Total	51,66	32,77
	NT	Male	39,86	20,88
		Female	55,86	24,87
		Total	47,86	23,98
Cells with one micronucleus	T	Male	23,03	14,30
		Female	28,10	14,97
		Total	25,56	14,61
	NT	Male	20,03	10,28
		Female	27,03	10,88
		Total	23,53	10,99
Cells with two micronuclei	T	Male	12,80	9,37
		Female	15,00	10,00
		Total	13,90	9,59
	NT	Male	10,76	6,95
		Female	14,90	7,60
		Total	12,83	7,46
Cells with three micronuclei	T	Male	11,46	9,93
		Female	12,93	10,66
		Total	12,20	10,15
	NT	Male	9,06	5,14
		Female	13,93	7,61
		Total	11,50	6,84

T - smokers; NT - non-smokers; DP - standard deviation.

There were no genotoxicity-related interactions among gender and smoking behavior, which differed from the prevalence of altered auditory efferent functions. There were, however, independent effects. Smokers had a significantly higher rate of DNA damage (as seen in the comet assay) compared to non-smokers (p=0.033). There was also a higher rate of genotoxicity (comet assay) in females (p=0,025), whether smokers or not.

Association among auditory efferent functions and genotoxicity indicators under a potential gender and smoking effect

There were no significant associations between absent DPOAE suppression, hearing difficulties in noisy environments, difficulty to understand speech in groups, discomfort with loud sounds, and genotoxicity.

Subjects that complained of hearing difficulties had a significantly higher rate of DNA damage (64.14±44.8) compared to subjects that did not present complaints of hearing difficulties (45.59±30.51) (p=0.049). This result did not depend on gender or smoking behavior. Self-reported tinnitus was also associated with a higher genotoxicity index (63.15±35.20) (p=0.027). As with hearing difficulty, this association was not influenced by gender or smoking behavior.

## DISCUSSION

Our results show that certain measures of auditory efferent pathway functions are affected by interactions among gender and smoking. Also, the complaints hearing difficulty and tinnitus may be associated with higher genotoxicity indices regardless of gender and smoking. These results are relevant because the study population consists of young normal-hearing adults (18 to 32 years).

As there are few studies on auditory efferent pathway functions and genotoxicity in young normal-hearing adults, the results will be discussed in greater detail below, starting with the estimated prevalence of findings.

In males, DPOAE suppression was present at a higher rate at 2000 Hz in left ears and at 6000 Hz in right ears only in male smokers. A higher rate of DPOAE suppression in male smokers may be explained by the presence of nicotine in cigarettes. A recent study^23^ has shown that the acetylcholine nicotinic receptors α9 and α10 are critical components of the medial olivocochlear system, and that the pharmacological properties of these receptors are similar to those of the cholinergic response of outer hair cells. These authors suggested that these receptors mediate auditory efferent fiber responses, accounting for cochlear efferent inhibition; this explains some of our findings in the present study. Other authors^24^ have investigated nicotinic receptors in auditory pathways and the role of cholinergic mechanisms in hearing; their results are consistent with the hypothesis that nicotinic cholinergic mechanisms exert a role in propagating auditory stimuli.

There were no significant differences between male smokers and non-smokers with regards to other auditory complaints related to the auditory efferent pathway that were studied here. These results could not be compared with other studies because we found no similar papers in the indexed literature on auditory efferent pathway function in smokers. It should be underlined that smoking affects hearing.^25^ Tobacco may affect hearing because of its effects on antioxidant processes or on blood vessels that irrigate auditory organs;^25^ tobacco also raises the level of carbon dioxide and nicotine.^9^ As a consequence, blood vessels may contract unduly and thrombotic occlusion may occur. Several studies have suggested that smoking is a significant risk factor for hearing loss;^25^, ^26^, ^27^, ^28^, ^29^ thus, it is relevant to study the effect of smoking on auditory efferent pathway function.

Female smokers complained mostly of difficulty to understand speech in noisy environments (Fig. 3). It is thought that this complaint is related with the auditory efferent system,^2^ which has an important role in protection against noise, location of sources of sound, and improved detection of sources of sound in noisy environments; no reports of smoking interfering with this function were found.

We found that smokers, regardless of gender, and females (smokers or not) had high genotoxicity indices; evidence were the statistically significant DNA damage indices (comet assay), which assesses genotoxicity caused by oxidative stress (Table 3). Other studies have also shown that smoking increases oxidative stress, resulting in genotoxicity,^6^, ^7^, ^8^ which may affect cell replication and transcription and cause cell death and/or cell mutations.

Other authors^30^ have also investigated the effect of smoking on oxidative damage to DNA in peripheral blood cells. These authors used 8-hydroxydeoxyguanosine (8-OH-dG) as a marker. In ten health male volunteers aged from 20 to 22 years, 5 ml of blood was taken before and 10 minutes after smoking two cigarettes within 10 minutes. The membrane of blood cells was lysed and DNA was taken from leukocytes with a DNA extractor; levels of 8-OH-dG were measured using high-performance liquid chromatography with electrochemical detection. The results revealed that mean 8-OH-dG levels increased significantly (p<0.05) from 3.3±0.8dG to 5.1±2.5dG after smoking. The authors concluded that smoking causes oxidative DNA damage in peripheral blood cells within a short time frame, which is similar to our findings in the present study.

A study in Colômbia,^8^ which was similar to the present study, evaluated genotoxicity due to tobacco exposure in youths. The authors^8^ assessed the frequency of chromosomal aberrations in peripheral blood lymphocytes of 32 young smokers and 32 young non-smokers aged from 19 to 29 years. The frequency of chromosomal aberrations was significantly higher in young smokers (6.02±0.52) compared to non-smokers (3.04±0.50); an association between genotoxicity and smoking was also shown.

With regards to interactions between genotoxicity and auditory efferent pathway function, only subjects with complaints of hearing difficulty and tinnitus, male or female smokers or not, had statistically significant higher genotoxicity indices. Other auditory efferent pathway functions were not positively associated with genotoxicity in our study subjects. We found no papers on the association between auditory efferent pathway function and genotoxicity due to oxidative stress; however, since oxidative stress occurs in any part of the body, it seems that it may also affect auditory efferent pathway function. A study relating oxidative stress to hearing^10^ suggested that oxidative stress could be an important factor in cochlear events, such as noise-induced sensorineural hearing loss or ototoxic drugs. Although reactive oxygen species - or oxygen free radicals - are normal byproducts of aerobic cell metabolism, these unstable molecules may destroy cell lipids, proteins, and DNA nucleic acids if the amount of antioxidants is compromised. The consequences of such rupture may be detected biochemically and histologically and demonstrated functionally. The abovementioned authors^10^ found that guinea-pigs exposed to noise with ensuing permanently altered auditory thresholds had measurable oxidative damage in cochlear DNA.

The results of the present study are relevant, notwithstanding the limitations of the method. These limitations are the small sample and being a cross-sectional study. However, initial exploratory studies, as was the case, are generally of this nature, with the purpose of revealing whether further studies with larger samples and a longitudinal or interventional design are needed. Thus, we believe that this study is relevant in auditory health and its disorders.

## CONCLUSION

Our results suggest that an association with oxidative stress, and more specifically with genotoxicity if interactions between gender and smoking are taken into account, may be observed in young normal-hearing adults with complaints about auditory efferent pathway functions, such as tinnitus and hearing difficulty. Additional studies are needed about the effect of other variables in this association.
